# Bacteremia Among Febrile Patients Attending Selected Healthcare Facilities in Ibadan, Nigeria

**DOI:** 10.1093/cid/ciz516

**Published:** 2019-10-30

**Authors:** Oluwafemi Popoola, Aderemi Kehinde, Veronica Ogunleye, Oluwafemi J Adewusi, Trevor Toy, Ondari D Mogeni, Eunice O Aroyewun, Sarah Agbi, Olukemi Adekanmbi, Akinlolu Adepoju, Sufiyan Muyibi, Ini Adebiyi, Oluseyi O Elaturoti, Chukwuemeka Nwimo, Hadizah Adeoti, Temitope Omotosho, Olabisi C Akinlabi, Paul A Adegoke, Olusoji A Adeyanju, Ursula Panzner, Stephen Baker, Se Eun Park, Florian Marks, Iruka N Okeke

**Affiliations:** 1 Department of Community Medicine, Faculty of Public Health, College of Medicine, University of Ibadan, Nigeria; 2 University College Hospital Ibadan, Nigeria; 3 Department of Medical Microbiology and Parasitology, and, Nigeria; 4 College of Medicine, University of Ibadan, Nigeria; 5 International Vaccine Institute, Seoul National University Research Park, Republic of Korea; 6 Department of Medicine, University of Ibadan; 7 Department of Paediatrics, College of Medicine, University of Ibadan; 8 Department of Pharmaceutical Microbiology, Faculty of Pharmacy, University of Ibadan; 9 Our Lady of Apostles Catholic Hospital, Oluyoro; 10 Adeoyo Maternity Teaching Hospital, Yemetu, Ibadan, Nigeria; 11 Department of Medicine, University of Cambridge, Cambridge, United Kingdom; 12 Hospital for Tropical Diseases, Wellcome Trust Major Overseas Programme, Oxford University Clinical Research Unit, Ho Chi Minh City, Vietnam

**Keywords:** bacteremia, *Salmonella*, malaria, antimicrobial resistance, Nigeria

## Abstract

**Background:**

The relative contribution of bacterial infections to febrile disease is poorly understood in many African countries due to diagnostic limitations. This study screened pediatric and adult patients attending 4 healthcare facilities in Ibadan, Nigeria, for bacteremia and malaria parasitemia.

**Methods:**

Febrile patients underwent clinical diagnosis, malaria parasite testing, and blood culture. Bacteria from positive blood cultures were isolated and speciated using biochemical and serological methods, and *Salmonella* subtyping was performed by polymerase chain reaction. Antimicrobial susceptibility was tested by disk diffusion.

**Results:**

A total of 682 patients were recruited between 16 June and 16 October 2017; 467 (68.5%) were <18 years of age. Bacterial pathogens were cultured from the blood of 117 (17.2%) patients, with *Staphylococcus aureus* (69 [59.0%]) and *Salmonella enterica* (34 [29.1%]) being the most common species recovered. Twenty-seven (79.4%) of the *Salmonella* isolates were serovar Typhi and the other 7 belonged to nontyphoidal *Salmonella* serovarieties. Thirty-four individuals were found to be coinfected with *Plasmodium falciparum* and bacteria. Five (14.7%) of these coinfections were with *Salmonella*, all in children aged <5 years. Antimicrobial susceptibility testing revealed that most of the *Salmonella* and *Staphylococcus* isolates were multidrug resistant.

**Conclusions:**

The study demonstrates that bacteria were commonly recovered from febrile patients with or without malaria in this location. Focused and extended epidemiological studies are needed for the introduction of typhoid conjugate vaccines that have the potential to prevent a major cause of severe community-acquired febrile diseases in our locality.

Community-acquired bacteremia causes considerable mortality and morbidity in low- and middle-income countries and most cases occur in children aged <5 years [1, 2]. Common causes of bacteremia in African countries include *Streptococcus pneumoniae*, *Staphylococcus aureus*, typhoidal and nontyphoidal *Salmonella enterica* serovars, *Haemophilus influenzae*, *Neisseria meningitidis*, *Klebsiella pneumoniae*, and *Escherichia coli* [1–5]. Patients with invasive bacterial infections commonly present with fever, a sign that is a root cause of medical visits, illnesses, and deaths in many tropical countries in Africa, including Nigeria [5, 6].

In Nigeria, there is considerable uncertainty about the main causes of fever [7]. The country is endemic for malaria and multiple fever-associated viruses. *Salmonella* Typhi is known to be endemic, although very little is known about the disease epidemiology. For example, the true cause of documented “epidemics” of typhoid fever between 1987 and 1989 was never proven to be *S*. Typhi bacteriologically, and these outbreaks may have been associated with *Plasmodium falciparum* malaria or other etiological agents [8]. In the following 3 decades, clinical diagnosis and the Widal test remain the predominant modes of typhoid diagnosis in Nigeria. Overestimation of typhoid fever incidence due to overreliance on the insufficiently specific Widal test is also common in other parts of Africa [7, 9, 10]. While there have been recent blood culture–based surveillance studies in other parts of Nigeria [4, 11], the true prevalence of invasive *Salmonella* in Ibadan, Nigeria, was undetermined at the start of this study.

In addition to doubts about the relative contribution of invasive pathogens to febrile disease in our setting, recent studies from elsewhere appear to suggest that these pathogens interact. Malaria may predispose individuals to nontyphoidal *Salmonella* (NTS) bacteremia by increasing iron availability to the pathogen (while depriving the host); in contrast, by incapacitating macrophage function, it is possible that bacteremia may also predispose susceptible individuals to malaria [4, 12, 13]. Altogether, the real and presumed high burdens of bacteremia and coinfection with malaria lead to patients in settings like ours receiving both antibacterials and antimalarials when they present with fever. This, in turn, increases the likely selective pressure for antimicrobial-resistant bacteria and plasmodia.

Determining the etiology of nonmalarial febrile illness remains challenging in Nigeria, even though malaria can be reliably diagnosed by microscopy or rapid diagnostic tests. Other leading causes of fever are not as easy to rule in or rule out due to limited diagnostic infrastructure [7, 14]. The near absence of a decision algorithm following a negative malaria test in most primary and secondary healthcare facilities compromises the potential diagnosis of nonmalarial febrile illness and increases unnecessary antimicrobial use [15, 16].

Typhoid fever and other bacteremias are best detected in clinical settings by blood culture, but serological alternatives exist as well [14]. The Widal test, for example, commonly used by African diagnosticians, can be performed with little skill and equipment and is cheap [10]. However, although the Widal test can support clinical diagnosis, it is characterized by nonspecificity, poor standardization, typical misuse, and often confusing results [7, 10, 17]. Blood culture services may circumvent many of these limitations but are slow and generally unavailable in low- and middle-income countries [18–20]. Blood culture is not used routinely in Nigerian hospitals, particularly in outpatients, and is rarely accessible outside tertiary care facilities. Even when blood culture facilities are available, they are often unaffordable for the majority of patients.

The World Health Organization has stressed the need for improved data on invasive *Salmonella* disease from countries in sub-Saharan Africa, with priority given to strengthening surveillance systems, including the surveillance of disease in children aged <15 years [21]. This study therefore aimed to profile bacteremia among febrile patients in selected hospitals in Ibadan, southwest Nigeria.

## METHODS

### Ethical Considerations

The study was approved by the University of Ibadan, University College Hospital (UCH) ethics review committee (approval number UI/EC/16/0389), the Oyo State Ethics Review Committee (approval number AD13/479/665A), and the Ethics Committee of Our Lady of Apostles Catholic Hospital, Ibadan (approval number OCH/EC/17/05). Written informed consent was provided by all participants or, in the case of children, their parents or guardians.

### Study Area

The study was conducted in Ibadan, southwest Nigeria, a large and ancient city that is now the administrative capital of Oyo state. Ibadan is delineated into a core municipal area and outlying metropolitan areas; the population of this area in the 2006 national census was 2 559 833. The data presented here cover the study-enrolled patients from 4 facilities between 16 June and 16 October 2017 at UCH (a tertiary facility); Our Lady of Apostles Catholic Hospital, Oluyoro; State Teaching Hospital, Adeoyo (secondary facility); and Kola Daisi Foundation Primary Health Care Centre (primary-level facility).

### Patients

Consecutive consenting patients who presented at the study sentinel healthcare facilities with objective fever of ≥38°C tympanic and/or ≥37.5°C axillary or at any of the facilities with fever reported for ≥3 consecutive days within the last 7 days or for whom the consulting clinician documented a clinical suspicion of typhoid fever were included in the study. All patients underwent full blood counts and blood culture, and the majority were tested for malaria parasites.

### Malaria Screening

Malaria screening was performed by microscopy of thin and thick films. One to 2 mL of blood was collected into an ethylenediaminetetraacetic acid bottle and agitated to avoid clotting. Next, 0.2 µL of blood was placed on one end of a clean slide for a thin film preparation while 0.6 µL was placed on the other end of the same slide for thick film. The smear was air-dried. The thin film was fixed with methanol, after which the slide was stained with a 1:10 dilution of freshly prepared Giemsa stain for 10 minutes. The stain was washed off with buffer water and air-dried. It was then examined under by light microscopy at ×1000 magnification.

### Blood Culture

Under aseptic conditions, blood was drawn from children and adults into a sterile syringe using a butterfly attachment or directly into a syringe and inoculated into a blood culture bottle (BACTEC). The importance of appropriate blood draws for blood culture, before initiation of antimicrobial treatment in healthcare facilities, where possible, and aseptic collection protocols were reinforced in training of study staff. One to 3 mL of blood was cultured for children and 8–10 mL in individuals >15 years of age, unless these volumes could not be collected, in which case the maximum volume collectable was cultured. Samples were transported to the laboratory immediately at room temperature.

At the laboratory, blood culture bottles were loaded into a BACTEC FX-40 (Becton Dickinson) continuously monitored blood culture instrument. Broth from positive bottles was examined directly by Gram stain and subcultured on solid culture media according to laboratory standard operating procedures. Gram-negative bacteria were identified using the Analytical Profile Index 20E (API-20E) and API-NH biochemical systems (bioMérieux). Other bacteria were identified using standard methods [22].

### *Salmonella* Confirmation and Subtyping

Presumptive *Salmonella* species were confirmed by polymerase chain reaction (PCR) using primers specific for the *invA* gene [23] and subtyped by PCR, according to the method of Tennant et al [24]. PCR reactions were constituted in Illustra PuRe Taq Ready-To-Go PCR Beads (GE Healthcare) and reactions were templated with boiled colonies. Table 1 lists the PCR primers that were used. Reactions were amplified in a Verti Thermocycler (Applied Biosystems) preceding cycling with an initial denaturation at 95°C for 2 minutes, and then performing 35 cycles of 95°C for 30 seconds, 55°C for 30 seconds, and 72°C for 2 minutes, followed by a terminal extension at 72°C for 5 minutes. Amplicons were resolved on 1.5% agarose gels, stained with ethidium bromide, and visualized using an ultraviolet transilluminator (UVP GelMax Imager).

**Table 1.** Oligonucleotide Primers Used for Polymerase Chain Reaction

*Salmonella* Serovariety	Serogroup	Target	Primer Name	Sequence 5′-3′	Amplicon Size	Reference
Generic *Salmonella*	…	*invA*	invA 139F	GTGAAATTATCGCCACGTTCGGGCAA	284	[23]
			invA 141R	TCATCGCACCGTCAAAGGAACC		
*S.* Enteritidis	Group D	*sdfI*	sdfF	TGTGTTTTATCTGATGCAAGAGG	333	[24]
			sdfR	CGTTCTTCTGGTACTTACGATGAC		
*S.* Dublin	Group D	*fliC*-g,p	H-for	ACTCAGGCTTCCCGTAACGC	779	[24]
			Hgp	ATTAACATCCGCCGCGCCAA		
*S.* Typhimurium	Group B	*fljB*	Sense-59	CAACAACAACCTGCAGCGTGTGCG	1389	[24]
			Antisense-83	GCCATATTTCAGCCTCTCGCCCG		
		*fliA/B*	FFLIB	CTGGCGACGATCTGTCGATG	250/1000	[25]
			RFLIA	GCGGTATACAGTGAATTCAC		
*S.* Paratyphi	Group B	SPAB_01124	pPB23-F	ACATAATGCTTTTCGTGCTCCTC	384	[24]
			pPB23-R	GGCATAAATATCTTTCTCCCCTCC		
*S.* Stanleyville	Group B	*fliC*- z24,z23	Hz4,z23F	TTTGTCAAAGATGTTACTGCG	427	[24]
			Hz4,z23R	AGGTTAGTGATGGCAGATTC		

### Antimicrobial Susceptibility Testing

Antimicrobial susceptibility testing was performed by the disk diffusion method according to Clinical and Laboratory Standards Institute (CLSI) guidelines [26]. *Salmonella* Typhimurium American Type Culture Collection (ATCC) 14028, *E. coli* ATCC 25922, and *S. aureus* ATCC 25923 were used as controls. Resistance data were analyzed in the 2018 version of WHONET [27].

## RESULTS

A total 682 patients drawn from all of the 11 Ibadan local government areas (LGAs) were enrolled in the study (Table 2). Seven of those recruited were infants aged <1 year and 467 (68.5%) were aged 1–17 years. Of those who were assessed by weight, 218 (82.6%) of pediatric and 15 (10.2%) of adult blood culture bottles were filled to the manufacturer-recommended volumes of 1–3 mL and 8–10 mL, respectively. Bacterial isolates were cultured from the blood of 169 (24.8%) of the recruited patients. Fifty-two (30.8%) of the recovered isolates were aerobic spore bearers and coagulase-negative staphylococci, which were considered contaminants such that 7.8% of blood cultures were contaminated. A probable bacterial pathogen was cultured from 117 (17.2%) patients, with *S. aureus* (69 [59.0%]) and *S. enterica* (34 [29.1%]) being the most common species identified (Table 3). Twenty-seven (79.4%) *Salmonella* species were largely *S.* Typhi, and 7 isolates belonging to NTS serovars were also recovered. No specimen yielded >1 likely bacterial pathogen.

**Table 2.** Descriptive Statistics of Febrile Patients Enrolled From 4 Ibadan Health Facilities During the Study

Variable	Frequency (%) (N = 682)
Age, y	
<1	7 (1.0)
1–5	217 (31.8)
6–12	189 (27.7)
13–17	61 (8.9)
18–59	198 (29.0)
≥60	10 (1.5)
Mean (SD)	15.2 (0.6)
Sex	
Male	332 (48.7)
Female	350 (51.3)
Site	
UCH	324 (47.5)
Oluyoro	141 (20.7)
Adeoyo	148 (21.7)
Kola Daisi Foundation Primary Health Centre	69 (10.1)
Presenting symptoms^a^	
Vomiting	312 (45.8)
Sore throat	279 (40.9)
Abdominal pain	245 (35.9)
Cough	192 (28.2)
Headache	179 (26.3)
Difficulty breathing	79 (11.6)
Residence location of recruited patients	
Ibadan outlying LGAs (n = 336)	
Ona-ara	68 (10.0)
Egbeda	83 (12.2)
Akinyele	40 (5.9)
Ido	61 (8.9)
Lagelu	55 (8.1)
Oluyole	29 (4.3)
Ibadan municipal LGAs (n = 342)	
Ibadan northwest	13 (1.9)
Ibadan southeast	18 (2.6)
Ibadan northeast	87 (12.8)
Ibadan north	191 (28.0)
Ibadan southwest	33 (4.8)
Outside Ibadan (n = 4)	4 (0.6)

Abbreviations: LGA, local government area; SD, standard deviation; UCH, University College Hospital Ibadan.

^a^Multiple responses.

**Table 3.** Pathogens Identified From Febrile Patients’ Blood Cultures

Probable Pathogens (n = 117)	No. (%) of Isolates Cultured From Enrolled Patients (N = 682)
*Staphylococcus aureus*	69 (10.1)
*Salmonella enterica* serovar Typhi	27 (4.0)
*Salmonella enterica* serovar Enteritidis	3 (0.4)
*Salmonella enterica* serovar Typhimurium	1 (0.1)
*Salmonella enterica*, other nontyphoidal	3 (0.4)
*Escherichia coli*	3 (0.4)
*Klebsiella pneumoniae*	2 (0.3)
*Citrobacter koseri*	1 (0.1)
*Enterobacter cloacae*	1 (0.1)
*Enterobacter aerogenes*	1 (0.1)
*Photobacterium damselae*	1 (0.1)
*Streptococcus pneumoniae*	1 (0.1)
*Haemophilus influenzae*	1 (0.1)
*Acinetobacter haemolyticus*	1 (0.1)
*Pseudomonas aeruginosa*	1 (0.1)
*Candida albicans*	1 (0.1)

Children aged 1–17 years exhibited the highest proportion of any bacterial and/or *Plasmodium* parasite infections. *Salmonella* Typhi was detected more than twice as frequently in children aged 6–12 years than in other age groups (Table 4); the proportion of malaria parasitemia per age-stratified recruited patients was similar across all age groups. Case numbers were much lower for NTS serovars, but 5 of the 7 NTS isolates were recovered from children aged <5 years.

**Table 4.** Detection of All Blood-cultured Bacteria, *Salmonella* Typhi, and *Plasmodium* Across the Different Age Groups

Age, y	Frequency (%) (N = 682)	All Cultured Bacteria, No. (% [95% CI])	*Salmonella* Typhi, No. (% [95% CI])	NTS, No. (% [95% CI])	Malaria, No. (% [95% CI])
<1	7 (1.0)	5 (0.7 [.3–1.6])	0 (0)	1 (0.2 [.0–.7])	3 (0.4 [.1–1.2])
1–5	217 (31.8)	39 (5.7 [4.2–7.7])	6 (0.9 [.4–1.8])	4 (0.6 [.2–1.4])	64 (9.4 [7.4–11.8])
6–12	189 (27.7)	38 (5.6 [4.0–7.5])	16 (2.4 [1.4–3.7])	1 (0.2 [.0–.7])	58 (8.5 [6.6–10.8])
13–17	61 (8.9)	11 (1.6 [.9–2.8])	3 (0.4 [.1–1.2])	1 (0.2 [.0–.7])	20 (2.9 [1.9–4.4])
18–59	198 (29.0)	22 (3.2 [2.0–4.8])	2 (0.3 [.1–1.0])	0 (0)	24 (3.5 [2.3–5.1])
≥60	10 (1.5)	1 (0.2 [.0–.7])	0 (0)	0 (0)	2 (0.3 [.1–1.2])

Abbreviations: CI, confidence interval; NTS, nontyphoidal *Salmonella*.

**Figure 1.**
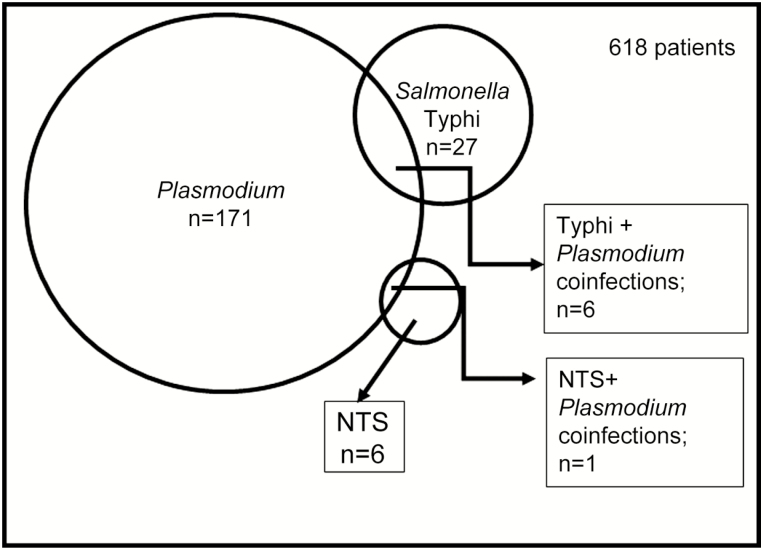
Detection of malaria parasites and *Salmonella* in blood from 618 patients. Malaria, *Salmonella* Typhi, and nontyphoidal *Salmonella* (NTS) were detected in 171, 27, and 6 patients, respectively. *Salmonella* Typhi/*Plasmodium falciparum* coinfection was detected in 4 patients and NTS/*Plasmodium* coinfection in 1 patient.

All 682 samples were blood cultured, and malaria testing was performed on all but for 64 of the 682 patients. Of the 618 patients tested for malaria, 171 (27.7%) tested positive, and *P. falciparum* was identified in every positive case. *Plasmodium falciparum* and bacterial coinfections were detected in 34 (5.5%) patients, which included malaria coinfection with 6 *S.* Typhi infections and 1 NTS infection (Figure 1). Malaria coinfection was also found in 24 (34.8%) of the patients infected with *S. aureus* and 1 of the 2 patients infected with *K. pneumoniae*, as well as the single patients from whom *Citrobacter koseri* and *Enterobacter cloacae* were isolated. Thirty (88.2%) patients coinfected with bacteria and malaria parasites were <18 years of age. All but 1 of the 7 patients with *Salmonella* and *Plasmodium* coinfection were children.

**Figure 2.**
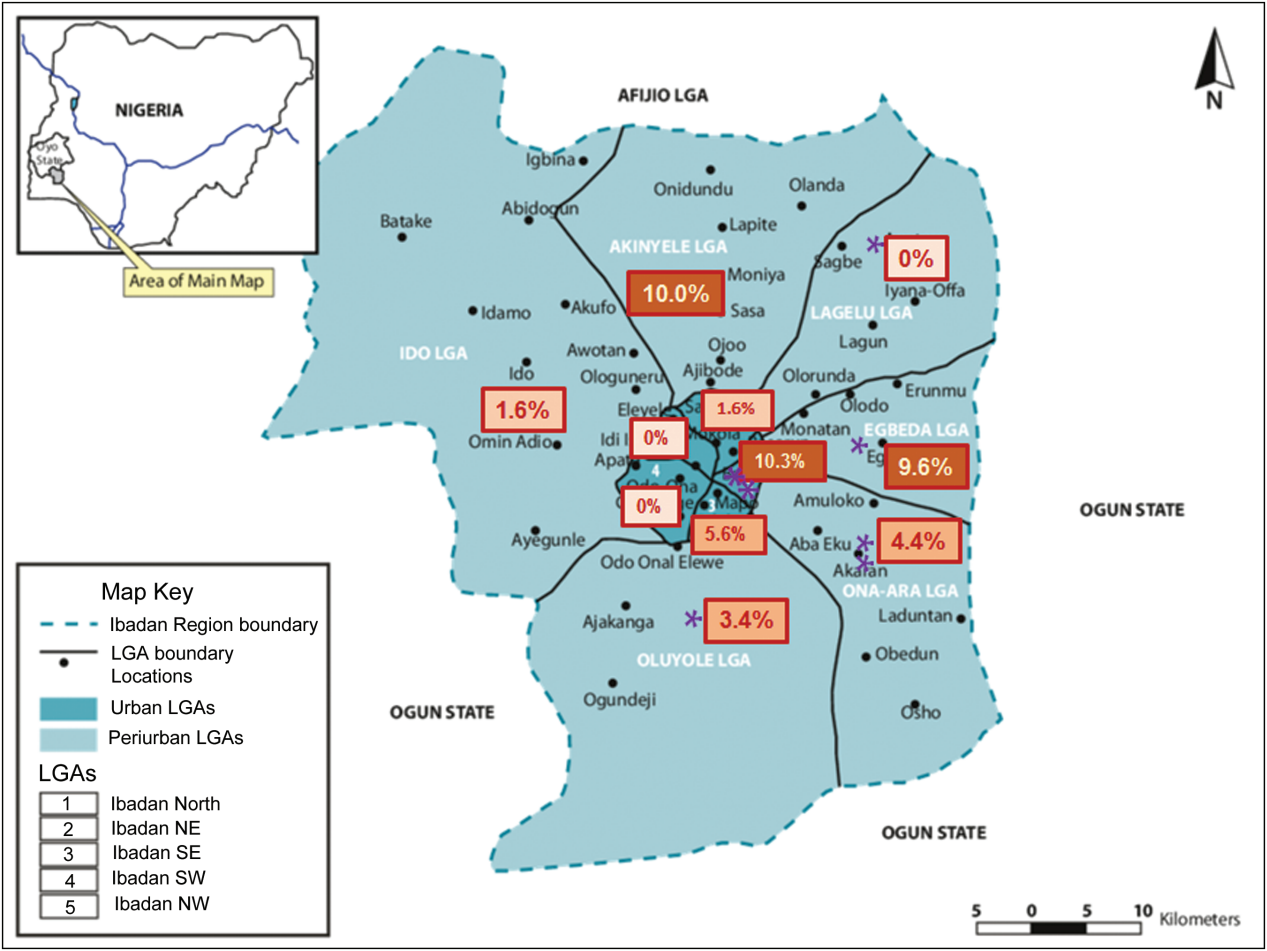
Recovery of *Salmonella* from outpatients visiting study facilities resident in the 5 urban municipal (dark teal) and 6 outlying (light teal) Ibadan local government areas (LGAs). Boxed numbers represent the percentage of participants residing in each Ibadan LGA from which *Salmonella* Typhi was cultured, and each purple asterisk represents a single *Salmonella* Paratyphi isolation. The base map for the figure was adapted from Adelekan et al [28]. Abbreviations: LGA, local government area; NE, northeast; NW, northwest; SE, southeast; SW, southwest.

We noted the residential LGAs for recruited patients to determine whether patients infected with 1 or more pathogens were predominantly drawn from specific sublocalities in Ibadan. The northeastern location of the study healthcare facilities predictably led to patient recruitment predominantly from northeast Ibadan (Table 2). However, as shown in Figure 2, patients enrolled in the study were drawn from all 11 LGAs, including the 6 outlying LGAs. *Salmonella* was cultured at higher frequencies from specific LGAs: *S.* Typhi recovery of from patients recruited from Akinyele, Ibadan northeast, and Egbeda LGAs was between 9.6% and 10.3%, whereas invasive *Salmonella* were detected at a prevalence of 0–1.6% in residents recruited from Ido, Ibadan north, Ibadan southwest, and Ibadan northwest (Figure 2). There was some, but not complete, overlap with high-recovery areas for *S.* Typhi and NTS.

Antimicrobial susceptibility testing revealed that all but 1 of the *Salmonella* isolates were resistant to at least 3 of the antimicrobial classes tested. Two *S.* Typhi and 3 invasive NTS (iNTS) isolates were resistant to 6 additional different agents. The median number of antimicrobials to which the *Salmonella*, both Typhi and iNTS, were resistant was 5 (Table 5). *Salmonella* Typhi and iNTS were universally resistant to ampicillin and most of the isolates were additionally resistant to tetracycline, trimethoprim-sulfamethoxazole, and nalidixic acid (Table 5). Nineteen (70.3%) of the *S.* Typhi isolates and 4 of the 7 NTS isolates were additionally ciprofloxacin nonsusceptible, demonstrating low-level ciprofloxacin resistance (classed as intermediate according to CLSI criteria). Resistance was similarly common among *S. aureus* isolates, with 64 (92.8%) being β-lactam resistant and 26 (37.7%) demonstrating resistance to cefoxitin (Table 6).

**Table 5.** Resistance Profiles of *Salmonella* Isolates

No. of Resistances	Resistance Profile	*Salmonella* Typhi	All iNTS	*Salmonella* Enteritidis	*Salmonella* Typhimurium
1	AMP	1	…	…	…
2	AMP, CXM	0	1	1	…
4	AMP, TCY, NAL, SXT	6	…	…	…
4	AMP, CIP, TCY, SXT	1	…	…	…
5	AMC, AMP, CHL, TCY, SXT	…	2	1	…
5	AMP, TCY, AZM, NAL, SXT	1	…	…	…
5	AMP, CIP, CXM, NAL, SXT	1	…	…	…
5	AMP, CIP, TCY, NAL, SXT	15	1	1	…
6	AMC, AMP, CHL, CIP, TCY, SXT	…	1	…	…
6	AMP, CHL, CIP, TCY, AZM, SXT	…	1	…	…
6	AMC, AMP, CIP, TCY, AZM, SXT	…	1	…	1
6	AMP, CIP, TCY, CXM, NAL, SXT	1	…	…	…
8	AMC, AMP, CIP, TCY, CXM, AZM, NAL, SXT	1	…	…	…

Abbreviations: AMC, amoxicillin/clavulanic acid; AMP, ampicillin; AZM,azithromycin; CIP, ciprofloxacin; CXM, cefuroxime; iNTS, invasive nontyphoidal *Salmonella*; NAL, nalidixic acid; SXT, trimethoprim-sulfamethoxazole; TCY, tetracycline.

**Table 6.** Antimicrobial Resistance of the *Staphylococcus aureus* Isolates

Antibacterial	No. of Strains Tested^a^	% Resistant	% Intermediate	% Sensitive
Ampicillin	69	92.8	0	7.2
Amoxicillin/clavulanic acid	65	44.6	0	55.4
Cefoxitin	69	37.7	0	62.3
Gentamicin	70	7.1	1.4	91.4
Ciprofloxacin	68	11.8	8.8	79.4
Clindamycin	67	10.4	7.5	82.1
Erythromycin	69	34.8	7.2	58
Chloramphenicol	69	20.3	2.9	76.8
Tetracycline	68	58.8	0	41.2

^a^Four isolates were not available for retesting and testing against antimicrobials for which data were missing.

## DISCUSSION

In this study, we performed blood culture and malaria parasite screening on specimens from febrile patients attending 4 healthcare facilities in Ibadan, Nigeria, and detected bacteria and/or plasmodia in 254 of the 618 (41.1%). In addition to our inability to perform malaria testing for 64 patients, particularly those recruited out of hours, there were other limitations to the study. Malaria parasitemia was detected by thick- and thin-film microscopy. More sensitive methods that measure parasite density are better suited to identifying clinical malaria in hyperendemic locations like ours but were not used in this study, nor did we collect data on malaria parasitemia in healthy individuals for comparison. Blood culture volumes were recorded for most, but not all, of the enrollees and a contamination rate of 7.8%, which could have obscured some infections, was recorded.

Febrile patients in Nigeria and other malaria-endemic countries are often presumed to have malaria and are managed accordingly. Limitations notwithstanding, this study confirms findings from other parts of Nigeria and the African continent, pointing to bacteremia as a principal cause of febrile illness, and the need for blood culture facilities and/or alternate diagnostics [2, 4, 6, 11, 29–31]. Bacteremia likely occurs more commonly than the 17.2% we report here because only a single aerobic culture was taken for each patient, which is known to have low sensitivity, particularly for typhoidal *Salmonella* [32, 33]. Additionally, antimicrobial pretreatment is common in our setting, with many patients having taken oral treatments before presenting at healthcare facilities. Antimicrobial use in the community will cause some patients with bacteremia to postpone seeking additional healthcare and will likely reduce pathogen recovery rate in those who do seek medical support. Blood culture sensitivity was potentially also reduced by contamination, even though a rate of 7.8% contamination is lower than recorded in most African studies [2, 4, 34].

*Salmonella* Typhi, an organism for which blood culture is only 40%–60% sensitive [32, 33], was isolated from 27 (4.0%) individuals and was the second most commonly isolated species/subspecies. Other common causes of bacteremia—NTS, *E. coli*, *S. pneumoniae*, *K. pneumoniae*, *Enterobacter aerogenes*, and *H. influenzae—*were less commonly recovered, and *Candida albicans* was detected in 1 positive culture. A similar study from more than a decade ago [2], which screened children admitted into a hospital in Kenya for bacteremia, found *E. coli*, *S. pneumoniae*, and *H. influenzae* to be the most predominant species recovered from children aged <5 years. In Lagos, Nigeria, a 2005 study of 74 patients with pyrexia of unknown origin identified *S.* Typhi (16.2%), *Proteus* species (14.9%), *E. coli* (12.2%), and *Klebsiella* species (9.5%), and also detected *Plasmodium* species in 44.6% of specimens [35]. A more recent study in Abuja, Nigeria, found *S. pneumoniae* to be the leading cause of death in children [4], and a second study in Abuja and Kano commonly recovered *S.* Typhi [11]. Similar spectra of pathogens have been recovered from studies in Ghana within the last decade [36].

Few studies in Nigeria have reported automated blood culture isolates from bacteremia patients, and those that have focused on children aged <5 years. In this important age group, the bacteremia recovery rates and range of pathogens in this study were remarkably similar to those recorded in Ibadan almost a decade previously [37]. A more recent retrospective survey of enteric fever in 3 large Nigerian cities (Abuja, Kano, and Lagos) reported *S*. Typhi detection rates ranging between 0.5% and 5% in Abuja (2009–2017) and between 4% and 10% in Kano (2013–2017) by blood culture [38]. These data parallel with the 4.8% (33/682) recorded in our 4-month Ibadan study. Although Lagos is physically closer to Ibadan than either of the other locations, data from Lagos (7%–19% between 1993 and 2015) were derived from diagnosis with the Widal test and are therefore not comparable with the current study. Our own bacteremia detection rates in children aged <5 years (21%) were slightly higher than those reported between 2008 and 2015 from Abuja in central Nigeria (7.5%) and Kano in northern Nigeria (15%), using a comparable automated blood culture methodology [11, 38]. The relative proportion of bacteremia attributable to *Salmonella* was lower (29.1%) in this study compared with the aforementioned Abuja study (60%), but comparable to the 24% recorded in Kano. Notably, *S.* Typhi predominated in all studies, but like previous investigations we also recovered a smaller number of NTS isolates, half (n = 3) of which were *Salmonella* Enteritidis. The approximately 4:1 ratio of *S.* Typhi to iNTS compared with a the ratio contained in recent report from northern Nigeria [11]. Altogether, although recovery rates of invasive *Salmonella* in this study are worrisome, they are at the lower end of recent reports from Nigeria, and differences in our data from a western Nigerian city point to possible within-country geographical differences in the etiology of community-acquired bacteremia.

Within our Ibadan study area, we recorded the residential LGA of the study participants. *Salmonella* Typhi was generally more commonly isolated from the blood of residents from the northern and eastern parts of Ibadan city. As shown in Figure 2, LGAs proximal to LGAs with high *S.* Typhi recovery showed intermediate recovery rates. Altogether, the data point to clustering of typhoidal infections, and as has been shown at other locations [39], spatial analysis of disease and reservoirs may help to pinpoint and reduce the risk of infection.

Malaria was diagnosed in 171 (27.7%) of the tested febrile patients in this study, including 6 of the patients with enteric fever. The 2003 report from Nsutebu et al from neighboring Cameroon [6] recorded only 3 (2.5%) patients with invasive *Salmonella* disease, whereas 94 (47%) had malaria and there were no coinfections [6]. A similar 2005 study in Lagos, Nigeria, which also did not record coinfections, recovered typhoidal *Salmonella* from 6.3% of the blood culture isolates and detected *Plasmodium* species in 44.6% [35]. Altogether, in contrast with earlier studies in malaria-endemic areas [5], co-detection of bacteremia and malaria parasites was common in this study. However, although the prevalence of coinfection was marked, the majority of patients do not require both antimalarials and antibacterials. Consequently, appropriate diagnostic support is likely to reduce the potential selective pressure on both categories of antimicrobials.

## CONCLUSIONS

In conclusion, we find that plasmodia, *S*. Typhi, and other bacteria are common in patients with community-acquired febrile disease in Ibadan, with both pathogens most commonly recovered from young children. The comparable clinical presentation of malaria and bacterial infections and limited patient overlap point to the need for laboratory verification of fever etiology to appropriately manage such infections. For this study, routine blood culture for febrile conditions, including those typically treated empirically, was implemented in a Nigerian hospital. While the time to diagnosis from blood culture is slow, the resulting etiologic information was extremely valuable. Rapid and cheaper tests for bacterial infections that can be implemented at nontertiary points of care and used parallel with malaria rapid diagnostic tests are urgently needed.

The high prevalence of *S.* Typhi infection detected in this study suggests that suspected typhoid needs to be rapidly and effectively treated and that even patients with laboratory-verified malaria infection should be screened for bacteremia. Interventions targeting these febrile diseases, such as vector control, water, sanitation, and hygiene, along with diagnostic test development and vaccine deployment need to be priorities in Nigeria. This study also creates a range of research questions, in particular source attribution and spatial risk for typhoidal *Salmonella* and the longitudinal trajectories for malaria and typhoid. These research areas can expand on this short study to measure the true burden of these diseases and the value of any future interventions.

## Notes

***Acknowledgments.*** The authors are grateful to the staff and administration of the 4 facilities where this study was performed; to project staff, in particular Adegoke Adeoye, Dr Olusegun K. Adepoju, R. Adetunji. Halima Babalola, Stephen O. Bejide, Veronica K. Dada, Abigail Kogsey, O. Ojo, Olubunmi Bola Oni, Olaide Olatoun, and Olayinka Raji; and International Vaccine Institute (IVI) team members Ji Hyun Han, Hyonjin Jeon, and Soo-Young Kwon for programmatic support and Anderson Oaikhena, Stella E. Ekpo, and Tu Le Thi for technical assistance.

***Financial support*.** This research was funded by the Bill & Melinda Gates Foundation [OPP1127988]. The International Vaccine Institute acknowledges its donors including the Republic of Korea and the Swedish International Development Cooperation Agency. This publication was made possible through a grant from the Bill & Melinda Gates Foundation [OPP1201031]. I. N. O. is supported by an African Research Leader Award from the UK Medical Research Council (MRC) and the UK Department for International Development (DFID) under the MRC/DFID Concordat agreement that is also part of the European and Developing Countries Clinical Trials Partnership 2 program supported by the European Union.

***Supplement sponsorship*.** This article was published as part of the supplement “Severe Typhoid Fever in Africa (SETA) Program” sponsored by the International Vaccine Institute.

***Potential conflicts of interest.*** All authors: No reported conflicts of interest. All authors have submitted the ICMJE Form for Disclosure of Potential Conflicts of Interest. Conflicts that the editors consider relevant to the content of the manuscript have been disclosed.

## References

[1] Hill PC, Onyeama CO, Ikumapayi UN, et al. Bacteraemia in patients admitted to an urban hospital in West Africa. BMC Infect Dis 2007; 7:2. 17257423 10.1186/1471-2334-7-2 PMC1794245

[2] Berkley JA, Lowe BS, Mwangi I, et al. Bacteremia among children admitted to a rural hospital in Kenya. N Engl J Med 2005; 352:39–47. 15635111 10.1056/NEJMoa040275

[3] Kohli R, Omuse G, Revathi G Antibacterial susceptibility patterns of blood stream isolates in patients investigated at the Aga Khan University Hospital, Nairobi. East Afr Med J 2010; 87:74–80. 23057259 10.4314/eamj.v87i2.60592

[4] Obaro S, Lawson L, Essen U, et al. Community acquired bacteremia in young children from central Nigeria—a pilot study. BMC Infect Dis 2011; 11:137. 21595963 10.1186/1471-2334-11-137 PMC3111365

[5] Evans JA, Adusei A, Timmann C, et al. High mortality of infant bacteraemia clinically indistinguishable from severe malaria. QJM 2004; 97:591–7. 15317928 10.1093/qjmed/hch093

[6] Nsutebu EF, Martins P, Adiogo D Prevalence of typhoid fever in febrile patients with symptoms clinically compatible with typhoid fever in Cameroon. Trop Med Int Health 2003; 8:575–8. 12791064 10.1046/j.1365-3156.2003.01012.x

[7] Okeke IN. Divining without seeds: the case for strengthening laboratory medicine in Africa. Ithaca, NY: ILR/Cornell University Press, 2011.

[8] Onuigbo MA Diagnosis of typhoid fever in Nigeria: misuse of the Widal test. Trans R Soc Trop Med Hyg 1990; 84:129–31. 2345914 10.1016/0035-9203(90)90404-3

[9] Kariuki S, Mwituria J, Munyalo A, Revathi G, Onsongo J Typhoid is over-reported in Embu and Nairobi, Kenya. Afr J Health Sci 2004; 11:103–10. 17298127

[10] Nsutebu EF, Ndumbe PM, Koulla S The increase in occurrence of typhoid fever in Cameroon: overdiagnosis due to misuse of the Widal test? Trans R Soc Trop Med Hyg 2002; 96:64–7. 11925996 10.1016/s0035-9203(02)90243-9

[11] Obaro SK, Hassan-Hanga F, Olateju EK, et al. *Salmonella* bacteremia among children in central and northwest Nigeria, 2008-2015. Clin Infect Dis 2015; 61(Suppl 4):S325–31. 26449948 10.1093/cid/civ745 PMC4596937

[12] Scott JA, Berkley JA, Mwangi I, et al. Relation between falciparum malaria and bacteraemia in Kenyan children: a population-based, case-control study and a longitudinal study. Lancet 2011; 378:1316–23. 21903251 10.1016/S0140-6736(11)60888-X PMC3192903

[13] Lokken KL, Stull-Lane AR, Poels K, Tsolis RM Malaria parasite-mediated alteration of macrophage function and increased iron availability predispose to disseminated non-typhoidal *Salmonella* infection. Infect Immun 2018; 86. doi:10.1128/IAI.00301-18. PMC6105891 29986892

[14] Baker S, Favorov M, Dougan G Searching for the elusive typhoid diagnostic. BMC Infect Dis 2010; 10:45. 20205702 10.1186/1471-2334-10-45 PMC2846943

[15] Burchett HE, Leurent B, Baiden F, et al. Improving prescribing practices with rapid diagnostic tests (RDTs): synthesis of 10 studies to explore reasons for variation in malaria RDT uptake and adherence. BMJ Open 2017; 7:e012973. 10.1136/bmjopen-2016-012973 PMC5353269 28274962

[16] Hopkins H, Bruxvoort KJ, Cairns ME, et al. Impact of introduction of rapid diagnostic tests for malaria on antibiotic prescribing: analysis of observational and randomised studies in public and private healthcare settings. BMJ 2017; 356:j1054. 28356302 10.1136/bmj.j1054 PMC5370398

[17] Otegbayo JA, Daramola OO, Onyegbutulem HC, Balogun WF, Oguntoye OO, Daramola OO Retrospective analysis of typhoid fever in a tropical tertiary health facility. Trop Gastroenterol 2002; 23:9–12. 12170927

[18] Penno EC, Baird SJ, Crump JA Cost-effectiveness of surveillance for bloodstream infections for sepsis management in low-resource settings. Am J Trop Med Hyg 2015; 93:850–60. 26175032 10.4269/ajtmh.15-0083 PMC4596611

[19] Okeke IN Diagnostic insufficiency in Africa. Clin Infect Dis 2006; 42:1501–3. 16619170 10.1086/503308

[20] Berkelman R, Cassell G, Specter S, Hamburg M, Klugman K The “Achilles heel” of global efforts to combat infectious diseases. Clin Infect Dis 2006; 42:1503–4. 10.1086/504494 16619171

[21] World Health Organization. Typhoid vaccines: WHO position paper. Geneva, Switzerland: WHO, 2008. 10.1016/j.vaccine.2018.04.022 29661581

[22] Cheesbrough M. District laboratory practice in tropical countries. 2nd ed. Cambridge, UK: Cambridge University Press, 2005.

[23] Rahn K, De Grandis SA, Clarke RC, et al. Amplification of an invA gene sequence of *Salmonella* Typhimurium by polymerase chain reaction as a specific method of detection of *Salmonella*. Mol Cell Probes 1992; 6:271–9. 1528198 10.1016/0890-8508(92)90002-f

[24] Tennant SM, Diallo S, Levy H, et al. Identification by PCR of non-typhoidal *Salmonella enterica* serovars associated with invasive infections among febrile patients in Mali. PLoS Negl Trop Dis 2010; 4:e621. 20231882 10.1371/journal.pntd.0000621 PMC2834738

[25] Garmendia J, Ren Z, Tennant S, et al. Distribution of tccP in clinical enterohemorrhagic and enteropathogenic *Escherichia coli* isolates. J Clin Microbiol 2005; 43:5715–20. 16272509 10.1128/JCM.43.11.5715-5720.2005 PMC1287796

[26] Clinical and Laboratory Standards Institute. Performance standards for antimicrobial susceptibility testing. Wayne, PA: CLSI, 2018:258.

[27] O’Brien TF, Stelling JM WHONET: an information system for monitoring antimicrobial resistance. Emerg Infect Dis 1995; 1:66. 10.3201/eid0102.950209 PMC2626837 8903165

[28] Adelekan I, Olajide-Taiwo L, Ayorinde A, Ajayi D, Babajide S. Building urban resilience: assessing urban and peri-urban agriculture in Ibadan, Nigeria. Padgham J, Jabbour J, eds. Nairobi, Kenya: United Nations Environment Programme, 2014.

[29] Makanjuola B, Bakare RA, Fayemiwo SA Quinolone and multidrug resistant *Salmonella* Typhi in Ibadan, Nigeria. Intern J Trop Med 2012; 7:103–7.

[30] Dekker D, Krumkamp R, Eibach D, et al. Characterization of *Salmonella enterica* from invasive bloodstream infections and water sources in rural Ghana. BMC Infect Dis 2018; 18:47. 29351771 10.1186/s12879-018-2957-4 PMC5775569

[31] Kariuki S, Revathi G, Kariuki N, Kiiru J, Mwituria J, Hart CA Characterisation of community acquired non-typhoidal *Salmonella* from bacteraemia and diarrhoeal infections in children admitted to hospital in Nairobi, Kenya. BMC Microbiol 2006; 6:101. 17173674 10.1186/1471-2180-6-101 PMC1764016

[32] Bell G, Gouyon PH Arming the enemy: the evolution of resistance to self-proteins. Microbiology 2003; 149:1367–75. 12777478 10.1099/mic.0.26265-0

[33] Gilman RH, Terminel M, Levine MM, Hernandez-Mendoza P, Hornick RB Relative efficacy of blood, urine, rectal swab, bone-marrow, and rose-spot cultures for recovery of *Salmonella* Typhi in typhoid fever. Lancet 1975; 1:1211–3. 48834 10.1016/s0140-6736(75)92194-7

[34] Brent AJ, Ahmed I, Ndiritu M, et al. Incidence of clinically significant bacteraemia in children who present to hospital in Kenya: community-based observational study. Lancet 2006; 367:482–8. 16473125 10.1016/S0140-6736(06)68180-4

[35] Akinyemi KO, Smith SI, Oyefolu AO, Coker AO Multidrug resistance in *Salmonella enterica* serovar Typhi isolated from patients with typhoid fever complications in Lagos, Nigeria. Public Health 2005; 119:321–7. 15733694 10.1016/j.puhe.2004.04.009

[36] Obeng-Nkrumah N, Labi AK, Addison NO, Labi JE, Awuah-Mensah G Trends in paediatric and adult bloodstream infections at a Ghanaian referral hospital: a retrospective study. Ann Clin Microbiol Antimicrob 2016; 15:49. 27539221 10.1186/s12941-016-0163-z PMC4991019

[37] Falade AG, Lagunju IA, Bakare RA, Odekanmi AA, Adegbola RA Invasive pneumococcal disease in children aged <5 years admitted to 3 urban hospitals in Ibadan, Nigeria. Clin Infect Dis 2009; 48(Suppl 2):S190–6. 19191615 10.1086/596500

[38] Akinyemi KO, Oyefolu AOB, Mutiu WB, et al. Typhoid fever: tracking the trend in Nigeria. Am J Trop Med Hyg 2018; 99:41–7. 30047362 10.4269/ajtmh.18-0045 PMC6128359

[39] Karkey A, Arjyal A, Anders KL, et al. The burden and characteristics of enteric fever at a healthcare facility in a densely populated area of Kathmandu. PLoS One 2010; 5:e13988. 21085575 10.1371/journal.pone.0013988 PMC2981554

